# The consequences of pain in early life: injury-induced plasticity in developing pain pathways

**DOI:** 10.1111/ejn.12414

**Published:** 2014-02-04

**Authors:** Fred Schwaller, Maria Fitzgerald

**Affiliations:** 1 Department of Neuroscience, Physiology & Pharmacology, University College LondonLondon, UK

**Keywords:** cannabinoids, descending pain control, experience dependent-plasticity, hyperalgesia, newborn infant

## Abstract

Pain in infancy influences pain reactivity in later life, but how and why this occurs is poorly understood. Here we review the evidence for developmental plasticity of nociceptive pathways in animal models and discuss the peripheral and central mechanisms that underlie this plasticity. Adults who have experienced neonatal injury display increased pain and injury-induced hyperalgesia in the affected region but mild injury can also induce widespread baseline hyposensitivity across the rest of the body surface, suggesting the involvement of several underlying mechanisms, depending upon the type of early life experience. Peripheral nerve sprouting and dorsal horn central sensitization, disinhibition and neuroimmune priming are discussed in relation to the increased pain and hyperalgesia, while altered descending pain control systems driven, in part, by changes in the stress/HPA axis are discussed in relation to the widespread hypoalgesia. Finally, it is proposed that the endocannabinoid system deserves further attention in the search for mechanisms underlying injury-induced changes in pain processing in infants and children.

## Introduction

Pain and injury in early life can cause lasting changes to developing somatosensory and pain systems. The immature nervous system in both humans and rodents is highly responsive to tactile and noxious stimulation; neurophysiological recordings reveal strong spinal nociceptive reflex activity and distinct nociceptive cortical potentials in response to clinically required skin-breaking procedures in newborn human infants (Slater *et al*., 2010b; Fabrizi *et al*., 2011; Cornelissen *et al*., 2013). This noxious evoked activity is more prolonged in the youngest infants and rat pups and decreases in duration with postnatal age (Fitzgerald & Gibson, 1984; Cornelissen *et al*., 2013). Importantly, when noxious stimulation in infancy is repeated or persistent, such as in neonatal intensive care or neonatal surgery, the effects outlast the period of stimulation itself and can result in profound and long-lasting changes in nociceptive neural pathways. By the time preterm infants reach term, they are already displaying enhanced cortical activity to an acute noxious stimulation compared with age-matched term-born controls (Slater *et al*., 2010a) and later in childhood these children display considerable alterations in somatosensory and pain processing (Hohmeister *et al*., 2009; Walker *et al*., 2009a). Taken together, studies show that both preterm and full-term children with neonatal intensive care unit (NICU) experience display greater perceptual sensitization to tonic heat (Hohmeister *et al*., 2009) accompanied by a generalized decreased sensitivity to cutaneous thermal (Walker *et al*., 2009a) and mechanical (Schmelzle-Lubiecki *et al*., 2007) stimulation. Specific injuries, such as moderate or severe burns in infancy, also result in greater pain and perceptual sensitization to noxious stimulation combined with depressed general mechanical and thermal sensitivity (Wollgarten-Hadamek *et al*., 2009). Severe infant burns are also associated with an attenuated social stress-induced analgesia, suggesting reduced function in phasic endogenous pain inhibitory mechanisms later in childhood and adolescence (Wollgarten-Hadamek *et al*., 2011). Major surgery within the first 3 months of life increases pain sensitivity and analgesic requirements to subsequent surgery compared with infants with no prior surgery (Peters *et al*., 2005). The different patterns of altered sensory and pain sensitivity in these studies may reflect differences in experienced pain, stress and analgesic treatment in these children but nevertheless it is clear that early painful injuries can induce local and global, long-term alterations in sensory and pain processing.

Long-term changes in pain processing in ex-preterm and early injured infants are likely to have several underlying causes. The number of tissue-breaking, presumed painful, procedures in neonatal intensive care is directly correlated with reduced brain white matter and subcortical grey matter (Brummelte *et al*., 2012) as well as delayed corticospinal development (Zwicker *et al*., 2013) and lower postnatal growth (Vinall *et al*., 2012), all with wide-ranging complex effects upon central nervous system (CNS) function. The aim of this review is to focus on the specific neurobiological mechanisms underlying injury-induced plasticity in the developing pain system. We summarize the main animal models in this field and review the evidence for activity-dependent cellular and synaptic changes in nociceptive circuitry in these models. We discuss two ways in which these changes may be maintained, namely neuroimmune activation and altered descending pain control, driven from the hypothalamic/pituitary/adrenal (HPA) axis. Finally, we suggest that the endocannabinoid system may be an interesting target for the prevention of injury-induced plasticity in the developing pain pathways.

## Rodent models of long-term effects of neonatal tissue injury

Understanding how pain and injury in early life can cause lasting changes to developing somatosensory and pain systems requires good animal models. A key requirement of such a model is that the effect is ‘age sensitive’ or has a ‘critical period’ such that the same injury applied in older or adult animals fails to have the same long-lasting effect on pain processing (Walker, 2013). For identification of neonatal critical periods in animal studies, appropriate controls in adulthood are crucial. The same degree and intensity of injury at different ages must be achievable to isolate specific developmental effects associated with injury or stress during a particular developmental period.

Table1 shows the different rodent models of long-term effects of neonatal injury upon pain processing. Many have been adapted from adult pain research and compare similar types and severities of injury in neonatal and adult rodents. These include: neonatal hindpaw plantar incision (Walker *et al*., 2009c), hindpaw inflammation with agents such as carrageenan (CAR) or complete Freund’s adjuvant (CFA) (Beland & Fitzgerald, 2001; Walker *et al*., 2003; Ren *et al*., 2004; Hohmann *et al*., 2005; LaPrairie & Murphy, 2007), full thickness skin wound (Reynolds & Fitzgerald, 1995; Beggs *et al*., 2012a), repeated needle prick (Anand *et al*., 1999; Knaepen *et al*., 2013), peripheral nerve injury (Howard *et al*., 2005; Vega-Avelaira *et al*., 2012) and visceral injury caused by distension or inflammation (Al-Chaer *et al*., 2000; Randich *et al*., 2006; Wang *et al*., 2008).

**Table 1:** Summary of animal models of long-term changes in pain and nociception following injury or insult in early life

Model	Neonatal injury	Effects on adult baseline sensitivity	Effects on adult response to re-injury	Sex differences	Controls
Surgical incision	Hindpaw plantar incision	Generalized baseline hyposensitivity (F. Schwaller & S.M. Walker, pers. comm.)	Re-incision: enhanced segmental mechanical and thermal hyperalgesia (Hathway *et al*., 2009b; Walker *et al*., [Bibr b94][Bibr b95]; Beggs *et al*., 2012b)	(Beggs *et al*., 2012b)	Brief neonatal handling & anaesthesia. Age-matched adults undergoing their first incision
Abdominal surgery	Laparotomy (P0)	Generalized baseline hyposensitivity (Sternberg *et al*., 2005)	N/A	?	Brief maternal separation, anaesthesia and saline injection (also have baseline hypoalgesia). ^*^Not compared with adult laparotomy
Skin wound	1 mm × 1 mm hindpaw skin removal	Behavioural hypersensitivity and *hyperinnervation at site of injured skin (Reynolds & Fitzgerald,* 1995; Beggs *et al*., 2012a)	N/A	?	Age-matched non-wounded animals ^*^Hyperinnervation, but not behaviour, compared with adult skin wound
Needle	Four needle pricks daily between P0 and P7	Thermal hyperalgesia (Anand *et al*., 1999)	Increased hypersensitivity after adult paw incision (Knaepen *et al*., 2013)	Hypersensivity to CFA injection in males only (Knaepen *et al*., 2013)	Neonatal tactile stimulation. ^*^No comparison made to adult needle pricks (Anand et al., 1999).
Inflammation	Hindpaw carageenan (CAR) or complete Freunds adjuvant (CFA) injection	Generalized baseline hyposensitivity (detectable after P34) (Ren *et al*., 2004).	Enhanced hyperalgesia after re-inflammation (Ren *et al*., 2004) *Increased capsaicin-evoked hyperalgesia (Hohmann et al*., 2005)	Exacerbated hyposensitivity in females (LaPrairie & Murphy, 2007)	Neonatal saline injection Age-matched adults undergoing their first CAR/CFA injection
Visceral distension & inflammation	Colorectal distension (CRD) or mustard oil application daily Bladder inflammation	Increased sensitivity to distension. Increased noxious heat responses in abdominal and paw skin (Al-Chaer *et al*., 2000; Wang *et al*., 2008) *No difference in thermal/mechanical threshold baseline response (Randich et al*., 2006). *Bladder hypersensitivity (DeBerry et al*., 2010)	Increased abdominal hyperalgesia following bladder inflammation with zymosan (Randich *et al*., 2006)	?	Brief neonatal handling. Age-matched adults undergoing their first bout of CRD
Nerve Injury	Spared nerve injury (SNI)	No initial neuropathic pain response (Howard *et al*., 2005). Hypersensitivity appears after P30 (Vega-Avelaira *et al*., 2012)	N/A	?	Sham surgery at P10 (thigh incision) Compared with adult animals undergoing nerve injury

Model	Neonatal stress	Effect on adult pain processing	Sex differences	Controls
Neonatal limited bedding (NLB)	Paucity of nesting material between P2 and P9	Reduced mechanical thresholds in skeletal muscle and nociceptors (Green *et al*., 2011). Enhanced hyperalgesia (Alvarez *et al*., 2013) *Prolonged prostaglandin E_2_-induced hyperalgesia (Green et al*., 2011)	?	Age-matched adults with standard neonatal bedding and care conditions
Lipopolysaccharide (LPS)/endotoxin	*Intraperitoneal* 0.1 mg/kg LPS at P14 (Boissé *et al*., 2005).	Enhanced pain behaviour after adult LPS (Boissé *et al*., 2005)	None (Boissé *et al*., 2005)	Neonatal saline injection. Age-matched adults undergoing their first LPS injection

**Figure 1:**
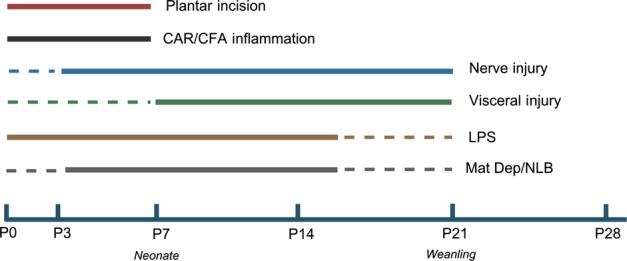
Critical periods for neonatal insults to cause long-term changes to pain and somatosensation in rodents. Solid lines indicate periods defined in controlled studies. Dotted lines indicate periods where data are incomplete. Postnatal age (P) is in days. CAR, carageenan; CFA, complete Freund’s adjuvant; LPS, lipopolysaccharide; Mat Dep, maternal deprivation; NLB, neonatal limited bedding. See text for further details.

**Figure 2:**
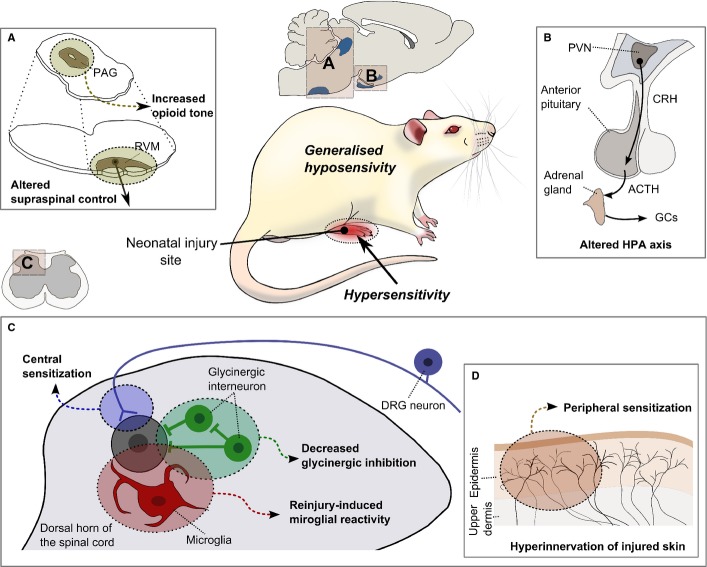
Potential mechanisms underlying the long-term effects of neonatal injury in adulthood. Changes to (A) brainstem descending pain control and (B) the HPA axis are hypothesized to underlie generalized baseline hyposensitivity whereas changes to (C) dorsal horn circuits and microglial reactivity and (D) peripheral terminals are hypothesized to underlie hypersensitivity in the region in and around the neonatal injury. See text for further details.

A common feature of these models is that a tissue injury at a critical period of development has long-term effects, outlasting the injury itself, resulting in adults with altered pain sensitivity compared with controls (Fig.1). The difference in pain sensitivity is assessed when the animals are adults as (1) changes in baseline sensory and nociceptive sensitivity as compared with controls (handled in exactly the same way as neonates but with no injury) and (2) changes in pain sensitivity (hyperalgesia) to re-injury as adults, compared with controls (adults receiving their first injury). This approach reveals a dual long-term effect of mild injury to the hindpaw (CAR inflammation or surgical incision) administered in the first 7–10 days of life. Mild injuries are associated with a widespread whole body baseline depression in sensory and nociceptive thresholds, or *hyposensitivity,* that emerges only when the rat is adolescent, i.e. 4–5 weeks old (Ren *et al*., 2004; Sternberg *et al*., 2005; Laprairie & Murphy, 2009). However, the area in and around the site of the neonatal injury retains an enhanced sensitivity to pain, so that a new injury applied to the region results in enhanced *hyperalgesia* that is greater in amplitude and more prolonged than controls (Ren *et al*., 2004; Chu *et al*., 2007; Walker *et al*., 2009b; Beggs *et al*., 2012b). The enhanced pain sensitivity can be observed within days of the first injury, but importantly is also present in the adult, long after the original neonatal injury has resolved. Neither the basal hyposensitivity nor the enhanced re-injury-associated hyperalgesia subside with age and are still evident in 120–125-day-old rats (Ren *et al*., 2004). Importantly, none of these effects occurs if the early inflammation or skin incision is administered after the first 7–10 days of life (Fig.2).

More extensive skin and subcutaneous, joint or visceral tissue injuries in early life also have long-term effects but are not associated with widespread hyposensitivity. These are characterized by a long-lasting mechanical sensitivity in and around the site of the neonatal injury, long after it has healed (Reynolds & Fitzgerald, 1995; Beggs *et al*., 2012a). This is especially striking after neonatal visceral injury, where repeated distension or mild inflammation in the first 2 weeks of life results in a long-lasting baseline visceral *hypersensitivity*, which also spreads outside the viscera to include the overlying abdominal and nearby cutaneous tissues (Al-Chaer *et al*., 2000; Randich *et al*., 2006; Wang *et al*., 2008; Christianson *et al*., 2010). Furthermore, when the animals are adults, a new visceral insult causes a greatly enhanced *hyperalgesia* compared with controls (Randich *et al*., 2006; DeBerry *et al*., 2010). Unlike milder cutaneous injuries, the critical period for these visceral effects is not constrained to the very early postnatal period (Fig.1). Thus, different types of injury may trigger different long-term effects in the somatosensory and pain system.

Peripheral nerve injuries in early life have a very different long-term effect on pain from neonatal tissue injury or inflammation. In the first postnatal days, the neurotrophin dependency of primary sensory neurons means that nerve injury causes substantial sensory neuron death (Huang & Reichardt, 2001) but even after this period, for the first 3 weeks of life little or no neuropathic pain results from nerve injury, in marked contrast to the effect in adults (Howard *et al*., 2005). However, later in life, when the animal reaches adolescence, nerve injury causes a distinct and persistent mechanical hypersensitivity (Vega-Avelaira *et al*., 2012).

The existence of two long-lasting changes in pain sensitivity after early tissue damage, increased pain and sensitivity to repeat injury on the one hand and widespread hypoalgesia on the other, each with different onset times, suggests distinct underlying mechanisms, some of which may lie outside the pain system (see Box ).

**Box 1**

**The influence of early life injury beyond pain pathways**

Many models of early life pain reveal changes to the CNS beyond the pain system. The overlap between pain and reward pathways (Borsook *et al*., 2007) suggests that neonatal pain experience may influence reward-related pathways and, in support of this, repeated neonatal skin incision does cause alterations in adult brain motivational orexinergic pathways, known to modulate mesolimbic dopaminergic reward circuitry, in an animal model of novelty-induced hypophagia (Low & Fitzgerald, 2012). Complex changes in alcohol preference are also observed following neonatal formalin and heel stick (Anand *et al*., 1999; Bhutta *et al*., 2001) and reduced exploratory activity following early visceral distension (Wang *et al*., 2008).

Neonatal pain has also been shown to influence the generation of new neurons in the dentate gyrus region of the hippocampus, thought to be involved in memory formation. Rat pups receiving intraplantar injections of the painful inflammatory agent complete Freund’s adjuvant, on P8, had more BrdU-labelled cells and a higher density of cells expressing doublecortin, both measures of newborn neurons, in the subgranular zone of the dentate gyrus (Leslie *et al*., 2011). In addition, neonatal paw inflammation causes long-term alterations to acute stress responses in adulthood, depending on the type of injury. A single intraplantar injection of carrageenan in the first postnatal week reduces adrenocorticotrophic hormone and glucocorticoid levels and reduces anxiety-like behaviours during the forced swim test in adulthood (Anseloni *et al*., 2005; Victoria *et al*., 2013) while hindpaw inflammation with complete Freund’s adjuvant and formalin increases anxiety-like behaviour in adults during elevated plus maze testing and forced swim tests (Roizenblatt *et al*., 2010; Mohamad *et al*., 2011; Negrigo *et al*., 2011).

## Mechanisms underlying the increased pain and hyperalgesia in the region of the neonatal injury

### The importance of neural activity from the original injury site

Increased pain and hyperalgesia following neonatal injury requires action potential activity in peripheral nerves supplying the injury site. Thus, treatment with local anaesthetics around the time of application of the injury prevents the long-term pain-enhancing effect (De Lima *et al*., 1999; Li *et al*., 2009; Walker *et al*., 2009b). Afferent input following neonatal injury is likely to be intense; continuous recording from single dorsal horn cells both before and after skin incision shows that the initial afferent-evoked spike activity is greater in young than in adult animals (Ririe *et al*., 2008).

### Sprouting and sensitization of peripheral nociceptors

Long-term pain hypersensitivity is associated with peripheral sensitization, especially where neonatal injury involves deeper or visceral tissue. Full thickness skin removal results in permanent changes in the innervation of the neonatally damaged region itself long after the wound has healed, leaving the area hyperinnervated by both myelinated A- and unmyelinated C-fibres (Reynolds & Fitzgerald, 1995; Beggs *et al*., 2012a). This hyperinnervation depends on the release of the neurotrophin NT-3 from the damaged region, which is highly up-regulated in the neonatal damaged area (Beggs *et al*., 2012a) combined with site-specific down-regulation of factors that normally inhibit axonal growth into the skin (Moss *et al*., 2005).

Long-term changes in afferent activity have been reported in adults following neonatal colon irritation. The average threshold of activation of sensory afferents decreased and spontaneous activity and responses to distension increased (Lin & Al-Chaer, 2003), but it is not known if this is also the case following skin injury or inflammation.

### Increased sensitivity of dorsal horn nociceptive circuits

Early life injury causes changes in spinal cord nociceptive circuitry similar to classic central sensitization or long-term potentiation. Thus, the enhanced pain sensitivity following neonatal skin wounds is accompanied by increased spinal flexion reflex electromyographic excitability (Beggs *et al*., 2012b) and enlarged dorsal horn receptive field areas (2.5-fold) in the region of damage, 6 weeks later (Al-Chaer *et al*., 2000; Torsney & Fitzgerald, 2003). These changes, where tested, are *N*-methyl-d-aspartate receptor dependent (Chu *et al*., 2007; Vega-Avelaira *et al*., 2012). Interestingly, visceral injury causes a spread of central sensitization that includes cutaneous inputs (Wang *et al*., 2008) due to the convergence of visceral and cutaneous inputs onto dorsal horn cells (Al-Chaer *et al*., 2000). Prolonged effects on synaptic signalling within adult spinal nociceptive circuits are likely to underlie this increased excitability: neonatal hindlimb skin and muscle tissue injury selectively increases the frequency, but not amplitude, of glutamatergic miniature excitatory postsynaptic currents (mEPSCs) in dorsal horn lamina II, recorded 2–3 days after injury, without altering miniature inhibitory postsynaptic currents (mIPSCs). This is followed by a persistent reduction in mEPSC frequency by 9–10 days post-injury (Li *et al*., 2009). When the animal reaches adulthood, a decrease in phasic inhibitory signalling is observed, due to a selective reduction in glycine receptor (GlyR)-mediated input to GABAergic and glutamatergic lamina II neurons and a decreased density of tonic GlyR-mediated current in the glutamatergic population (Li *et al*., 2013). The postnatal maturation of dorsal horn glycinergic circuits is dependent upon C-fibre activity in the first weeks of life (Koch *et al*., 2012) and this may explain the vulnerability of glycinergic neurons to alterations in nociceptive input during a critical developmental period (Koch & Fitzgerald, 2013).

In adults, there is good evidence that central sensitization contributes to many prolonged chronic pain states (Woolf, 2011), although in some cases, such as fibromyalgia (Staud *et al*., 2009), the pain requires a continuous peripheral input. However, the developmental plasticity under discussion here does not result in a ‘state’ of pain, but rather an increased hyperalgesia, which is a key feature of central sensitization, ‘an amplification of neural signaling within the CNS that elicits pain hypersensitivity’ (Woolf, 2011). The barrage of sensory input to the developing spinal cord that follows even a brief inflammation in early life results in significant changes in gene expression within the adult dorsal horn (Ren *et al*., 2005). The baseline condition in neonatally injured adult animals is characterized by up-regulation of γ-aminobutyric acid (GABA), cholecystokinin, histamine, serotonin and neurotensin systems in the dorsal horn ipsilateral to the neonatally injured paw. These changes in gene expression may lead to subthreshold increases in postsynaptic excitability that are unmasked by re-injury later in life. Epigenetic changes, i.e. chemical modifications of chromatin that modulate gene activity without altering the DNA sequence, are also likely to play a key role in this process, but little is known about this in relation to pain (Géranton, 2012).

### The role of neuroimmune activation

The long-term enhancement of pain activity that follows early injury might be maintained by the neuroimmune system. Glial activation in the peripheral and central nervous systems is a common characteristic of adult tissue and nerve injury (Scholz & Woolf, 2007) and microglial activation in specific regions of dorsal horn circuitry play a key role in central sensitization and hyperalgesia, through the release of cytokines and growth factors which excite nociceptive dorsal horn neurons (Trang *et al*., 2011). Strong C-fibre input into the adult spinal dorsal horn is sufficient to activate microglia and increase nociceptive reflexes (Hathway *et al*., 2009b). While C-fibre and tissue injury-induced activation of spinal microglia in young animals is much reduced compared with adults (Moss *et al*., 2007; Costigan *et al*., 2009), the immune system undergoes considerable postnatal maturation and may be ‘primed’ by early injury such that it is more easily activated in later life. This is supported by the fact that early-life immune activation such as lipopolysaccharide (LPS) causes enhanced baseline nociception and elevated basal spinal cord COX-2 compared with neonatally saline-treated rats (Boissé *et al*., 2005).

Adult plantar incision activates spinal microglia in the ipsilateral medial dorsal horn (Wen *et al*., 2009) but neonatal skin incision enhances the degree, distribution and time course of this microglial reactivity when the same hindpaw is subsequently re-incised in adulthood (Beggs *et al*., 2012b). Moreover, intrathecal injection of the microglial inhibitor minocycline at the time of adult re-incision prevents both microglial reactivity and enhanced hyperalgesia associated with the prior neonatal incision (Beggs *et al*., 2012b). As spinal microglia are an important source of pro-inflammatory cytokine synthesis and release after tissue damage, this neonatal ‘priming’ of microglia may increase the pro-inflammatory response of these cells after re-injury, enhancing spinal dorsal horn network excitability and behavioural hyperalgesia. Importantly, microglial ‘priming’ can be demonstrated in response to direct peripheral nerve C-fibre stimulation, bypassing the peripheral target tissue, in neonatally injured adults (Beggs *et al*., 2012b), showing that the injury-induced changes are maintained centrally in dorsal horn synapses and circuits. Later changes in the developing neuroimmune system at 3–4 weeks are also likely to underlie the delayed onset hypersensitivity that occurs following neonatal nerve injury (Costigan *et al*., 2009; Vega-Avelaira *et al*., 2012).

## Mechanisms underlying the widespread hypoalgesia following neonatal injury

The global reduction in baseline sensitivity following early life tissue damage must involve mechanisms beyond the highly somatotopically organized nociceptive pathways of the dorsal horn. In some models hypoalgesia is the dominating effect (Sternberg *et al*., 2005), whereas in others it is not a feature at all (Al-Chaer *et al*., 2000); but in many models, hypoalgesia forms a widespread backdrop to local increases in pain sensitivity (Ren *et al*., 2004).

### Altered brainstem descending pain control systems

The relatively delayed appearance of global hypoalgesia following neonatal injury coincides with the maturation of the descending pathways from the brainstem (Hathway *et al*., 2009a, 2012), raising the possibility that long-term changes in supraspinal circuitry occur after early injury. The brainstem rostroventral medulla (RVM) receives input from the periaqueductal grey (PAG), which in turn is driven from the amygdala and other limbic areas, and provides top-down control of several processes including nociception, as and when it is needed (Heinricher *et al*., 2009; Hellman & Mason, 2012). The RVM undergoes a maturational switch in the fourth postnatal week such that its control over spinal nociceptive circuits switches from being facilitatory before postnatal day (P) 21 to a gradually dominating inhibition between P25 and P40 (Hathway *et al*., 2009a, 2012). Blockade of tonic CNS opioidergic activity over the critical period of development (P21–P28) prevents the normal development of descending RVM inhibitory control of spinal nociceptive reflexes, while exogenous application of opioids accelerates it (Hathway *et al*., 2012).

There is evidence in animal models that descending pain control systems from the brainstem are altered by early pain experience. Focal electrical stimulation of the RVM in adult animals treated neonatally with CAR produced significantly greater descending inhibition of nociceptive responses to noxious thermal stimuli (Zhang *et al*., 2010). The mechanisms for this are not known but could involve alterations in peptidergic, 5-HT or GABA signalling in the different subclasses of RVM neurons. Significant postnatal developmental changes occur in opioidergic receptor expression in the PAG (Kwok *et al*., 2013) and inflammatory pain induced by intraplantar CAR at birth leads to increased beta-endorphin and met/leu-enkephalin protein levels and decreased opioid receptor expression in the adult PAG (LaPrairie & Murphy, 2009). Thus, a permanent change in RVM circuitry or neurotransmitter/receptor signalling as a result of early injury may alter the balance of descending control over spinal pain networks.

### Involvement of the HPA axis in hypoalgesia

It has been suggested the long-lasting changes in pain behaviour that follow early exposure to noxious stimuli may involve alterations in the stress HPA axis (Sternberg *et al*., 2005). While most studies make every attempt to control for handling and maternal separation (see Table1), noxious stimuli are inherent stressors. Infant stress is a strong indicator of short- and long-term alterations in brain function (Meaney *et al*., 1988; Papaioannou *et al*., 2002; Moriceau *et al*., 2009) although the developmental periods at which the human brain is sensitive to environmental stressors are not known (Pryce, 2008; Pechtel & Pizzagalli, 2011). Early life stress alone (with no noxious stimulation), such as those that target dam–pup interactions and feeding and nesting behaviours, can alter pain behaviour in later life (Table1). Neonatal maternal separation reduces sensitivity to noxious heat stimuli in adulthood compared with neonatally handled adults (Weaver *et al*., 2007) and induces visceral hypersensitivity in adult rats (Coutinho *et al*., 2002; Gosselin *et al*., 2010). Early life stress from a paucity of nesting material significantly prolongs muscle hyperalgesia following prostaglandin administration in the adult and increases the excitability of mature nociceptors innervating the muscle (Green *et al*. 2011). Restriction of dam and litter nesting material also increases plasma levels of the pro-inflammatory cytokine interleukin-6 in adulthood (Alvarez *et al*., 2013).

The mechanisms of the early life stress effect on pain pathways are not known but are probably mediated by the HPA axis, which in adults can directly influence the neurophysiological mechanisms underlying the perception of pain via brainstem descending pain control pathways (Butler & Finn, 2009). The HPA axis in rodents is relatively insensitive to noxious stimulation during the first 2 weeks of life, as long as maternal care is maintained (Levine, 1994, 2002; Lupien *et al*., 2009), but in preterm infants there is good evidence that intensive care procedures cause increases in plasma and salivary cortisol levels and in heart rate variability (Faye *et al*., 2010; Davis *et al*., 2011) that may be altered over longer periods (Grunau *et al*., 2010). It is not known whether this stress response directly influences the development of pain pathways in humans, but the immaturity of brainstem descending pain control pathways at birth (Hathway *et al*., 2009a, 2012) suggests that it could be highly modified by increased HPA activity.

The situation is complicated by the fact that development of the HPA axis itself is susceptible to a wide range of environmental, endocrine and immune stressors, some of which are also likely to be activated following neonatal injury (see Box). Important, too, is the other major stress axis, the sympathoadrenal system and its major mediator, the catecholamines, which have been shown to have a role in inducing and maintaining stress-induced enhancement of mechanical hyperalgesia in adults (Khasar *et al*., 2009).

## Cannabinoids and long-term plasticity of pain pathways

Agents aiming to reduce the long-term effects of early injury in infants and children would ideally combine analgesia with protection against physiological stress and immune activation. The cannabinoids are primarily analgesic, via CB1 receptors expressed in peripheral and central pain pathways modulating GABAergic and glutamatergic neurotransmission (Rea *et al*., 2007) and potentiating GlyRs (Xiong *et al*., 2011). They have strong immunomodulatory properties mainly mediated by CB2 receptors localized on immune cells (Guindon & Hohmann, 2009).

The endocannabinoid system is well developed at birth and high levels of CB1 expression in the human infant cortex suggest strong endocannabinoid regulation of presynaptic neurotransmission in the first few years of human life (Long *et al*., 2012). The system has a number of important functional roles in the neonatal brain, but from the perspective of this discussion the participation of endocannabinoids in the rapid suppression of neonatal physiological stress by glucocorticoids should be noted (Buwembo *et al*., 2012). Endocannabinoids are neuroprotective against neonatal sciatic nerve injury-induced cell death in neonatal rats (Perez *et al*., 2013) but it is not known if they could also protect against injury-induced plasticity in developing pain pathways. Importantly, the developmental trajectory of this system is sensitive to early life experience. Maternal deprivation reduces endocannabinoid ligand and receptor expression in the brain (Suárez *et al*., 2010) and neonatal LPS selectively down-regulates CB1 receptor expression in the amygdala (Zavitsanou *et al*., 2013). Conversely, CB1 receptor expression in the brain is significantly up-regulated by neonatal capsaicin, a C nociceptor toxin (Zavitsanou *et al*., 2010). Further exploration of the role of endocannabinoids in the developmental plasticity of pain pathways would be of great interest.

**Box 1**

**Neonatal environmental, endocrine and immune stressors and the developing HPA axis**

Stress activates the HPA axis: secretion of corticotropin-releasing hormone (CRH) from the hypothalamic paraventricular nucleus (PVN) controls adrenocorticotrophic hormone (ACTH) release from the pituitary which, in turn, regulates release of adrenal glucocorticoids (GCs), such as cortisol in humans or corticotropin (CORT) in rodents, which modulate physiological stress responses in a transient and reversible manner. Exposure to stress in neonatal rodents ‘programmes’ the HPA axis and other areas of the brain (Lupien *et al*., 2009). A critical developmental period of reduced responsiveness to stressful stimuli between P3 and P14 in rodents is maintained by maternal presence, which keeps levels of GCs low and is essential for normal neural and behavioural maturation (Sapolsky & Meaney, 1986; Walker & Vrana, 1993; Levine, 2002). Increasing GC levels during this period either by exogenous administration of GCs or by abnormal early life stress causes long-term changes to neuroendocrine and behavioural responses to future stressors (Plotsky & Meaney, 1993; Levine, 2002). More active maternal care during neonatal life is associated with reduced HPA-axis activation and behavioural responses to stressors in adulthood (Liu *et al*., 1997; Macrì *et al*., 2008) while maternal deprivation is associated with HPA-axis hyperactivity caused by increased CRH mRNA levels and CRH transcription in the PVN (Plotsky & Meaney, 1993; Chen *et al*., 2012). In addition to maternal grooming, other factors such as altered feeding patterns and temperature homeostasis during maternal separation alter HPA-axis responses later in life (Rüedi-Bettschen *et al*., 2004; Spencer, 2013). Neonatal endocrine and immune system interactions are also important for establishing stress responses later in life. Neonatal exposure to LPS increases HPA-axis responses to stress and to subsequent immune challenges in adulthood and are associated with an anxiety-like phenotype in adult rats which can be inherited by untreated offspring of the neonatally LPS-treated rats (Spencer *et al*., 2006; Walker *et al*., 2009a, 2012).

## Future perspectives

There is much still to understand in this important field of pain research. Future efforts should focus upon clarifying how the type, severity and site of injury affects the adult phenotype and the relative importance of stress vs. pain in this process. Equally important is to move beyond the effect itself and focus upon the peripheral and central mechanisms underlying injury-induced plasticity in developing pain pathways.

## Abbreviations

CAR: carrageenan
CNS: central nervous system
GlyR: glycine receptor
HPA: hypothalamic/pituitary/adrenal
LPS: lipopolysaccharide
PAG: periaqueductal grey
RVM: rostroventral medulla
